# Analytic Hierarchy Process-Based Construction Material Selection for Performance Improvement of Building Construction: The Case of a Concrete System Form

**DOI:** 10.3390/ma13071738

**Published:** 2020-04-08

**Authors:** Dongmin Lee, Dongyoun Lee, Myungdo Lee, Minju Kim, Taehoon Kim

**Affiliations:** 1 School of Civil, Environmental and Architectural Engineering, Korea University, 145, Anam-ro, Seongbuk-gu, Seoul 02841, Korea; ldm1230@korea.ac.kr (D.L.); dy_lee@korea.ac.kr (D.L.); Minju830@korea.ac.kr (M.K.); 2 Research and Development Center, Yunwoo Technology Co. Ltd., 128, Beobwon-ro, Songpa-gu, Seoul 058054, Korea; md.lee@yunwoo.co.kr

**Keywords:** material selection, project performance, material property, analytic hierarchy process (AHP), building construction, concrete system form

## Abstract

Selecting the best materials that ensure maximum performance is crucial in the construction engineering design of any construction project. However, this is challenging and usually not properly considered because of the lack of systematic and scientific evaluation methods for the performance of materials. This paper proposes a new approach of selecting material to satisfy the performance goal of material designers in building constructions based on the analytic hierarchy process method. To validate the suggested model, a case study was conducted for a concrete system form, the performance of which is susceptible to its materials and has a strong effect on overall project productivity. The newly developed form comprising polymers and alloys showed that the proposed material selection model provided a better combination of materials, and the solution was technically more advanced and ensured better performance. This paper contributes to the body of knowledge by expanding the understanding of how construction material properties affect project performance and provides a guideline for material engineers to select the best-performing building materials while considering a performance goal.

## 1. Introduction

Material selection is one of the most important yet complex tasks encountered by construction engineers, because it is directly related to overall project performance (e.g., time, cost, and quality) [1]. Construction engineers must select the best-performing materials based on the mechanical (e.g., specific strength and elasticity modulus), functional (e.g., noise reduction, corrosion resistance, and nonadhesiveness), and physical (e.g., density, color, and thermal conductivity [TC]) properties of the materials in the selection process in association with cost [2,3]. These evaluation criteria are often in conflict with each other, because an optimal selection for one criterion could sacrifice other criteria [2]. Therefore, construction material selection should be conducted through a systematic decision-making process, investigating how each criterion has an impact on project performance.

Despite the importance of material selection in construction projects, in practice, it mainly has been conducted with a heuristic approach based on personal experience because of the lack of a systematic evaluation model and an ambiguous measuring criterion for considering the potential performance of materials. Previously, some research has provided building material selection models using various methodologies, such as the ranking and scoring method [4], analytical network process (ANP) and technique for order of preference by similarity to ideal solution (TOPSIS) [5], quality function deployment (QFD) [6], and fuzzy-extended analytic hierarchy process (AHP) [7]. Those models provide useful guidance for the multicriteria decision-making (MCDM) of construction materials. However, previous research on material selection in the construction industry has mainly focused on the life cycle cost and sustainability of materials, especially for completed buildings. In addition, the characteristics of construction material and how they affect the potential performance of products have not been studied thoroughly.

To fill the gap in existing research, this paper aims to develop a general material selection model to help researchers and practitioners select optimal materials for construction products or facilities in terms of the performance goal. The performance goal can be defined by a material designer considering the user’s requirements. A systematic procedure through translating, screening, and rating processes provides a guideline to approach the performance goal in a systematic and scientific manner. The AHP method is a key technique during a rating process that quantifies all the qualitative properties of the material in terms of performance goal in building construction projects.

The aim of this study is to establish a generalized material selection model for construction engineers and practitioners for easy application. We tested the model in a case study based on selecting formwork materials such as panels and frames. Formwork plays a key role in concrete building construction because formwork cost accounts for as much as 15% of the total construction cost and approximately 25% of duration in a reinforced concrete structure [8,9]. In addition, there are many formwork materials commercially available in the market, enabling the manufacture of a real prototype and mock-up product based on the result of the case study. The remainder of the paper is arranged as follows. Section 2 explains a material selection process for performance improvement. Section 3 shows a case study on a concrete form, and we have reviewed it in Section 4. Section 5 describes the results, and Section 6 discusses the results. Finally, Section 7 concludes the study entirely.

## 2. Material Selection Process for Construction Materials

### 2.1. Material Selection Process

**Figure 1:**
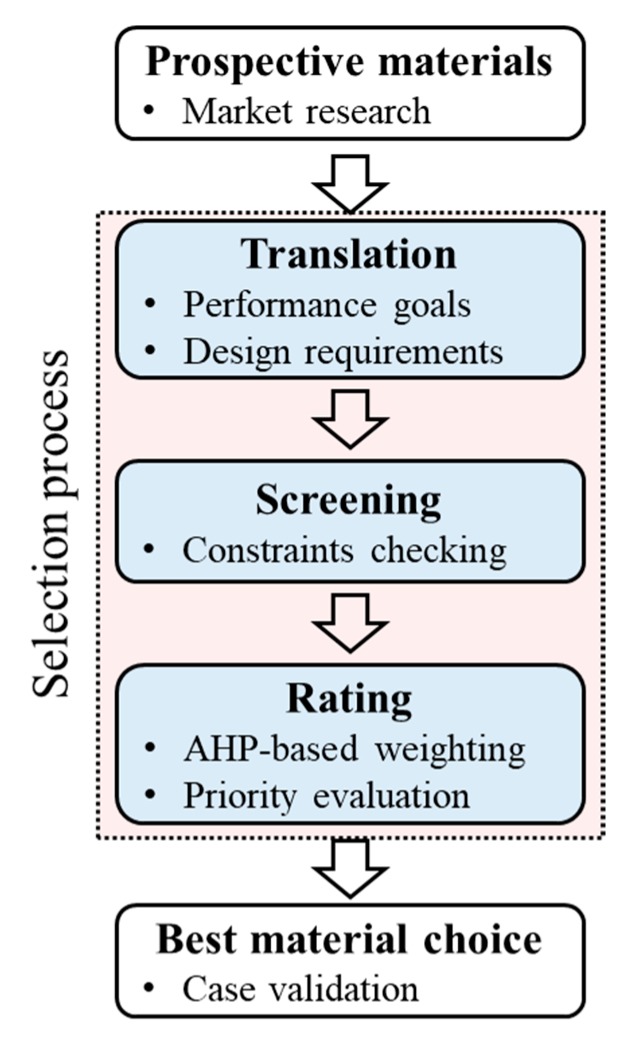
Material selection process for construction materials.

Material selection is one of the most significant and confusing tasks encountered by construction practitioners. In the construction materials market, there are 85,000 different commercially available polymers to choose from, at least 14 types of general-purpose engineering plastics, and at least 20 kinds of applicable alloys that are already commercialized. Available materials also may differ according to the country, market, and time, so the material engineer should consider allowable material candidates first before making a selection. The process is conducted in the order of translating, screening, and rating (Figure 1).

The first step for material selection is a translation. The material designer should define a technical performance goal (e.g., improved labor productivity and high maintainability). The performance goal should be testable and measurable like an index which is usually an accumulation of scores. The second step is a screening. Constraints (e.g., waterproof and high tensile strength) should be defined along with performance goals, and the constraints are used to eliminate ineligible candidates so we can save time for the rating process. The third step is a rating. Through this step, we evaluate what properties of the materials affect the performance goal. In this step, AHP is a key method that calculates the relative weight of material properties for maximizing the potential performance goal. AHP is a powerful yet simple method for making decisions even when important elements of the decision are difficult to quantify or compare.

### 2.2. Analytic Hierarchy Process (AHP)

**Figure 2:**
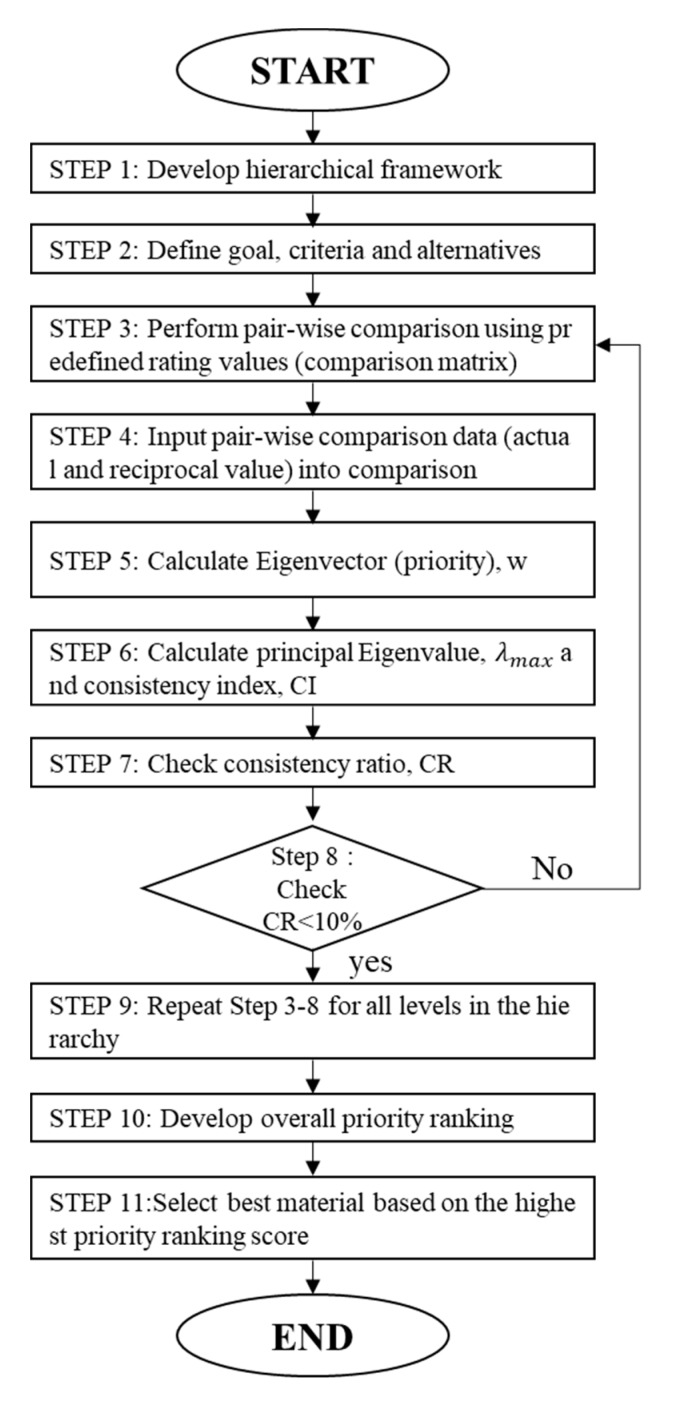
Analytic hierarchy process (AHP) methodology for material selection.

The AHP method, conceptualized by Saaty [10], is one of the most popular MCDM methods [11]. The purpose of MCDM is to select the best alternative from a set of competitive alternatives and evaluate it with a set of criteria [12]. The AHP method can be successfully applied to analyze qualitative data quantitatively. It transforms a complex and multicriteria problem into a structured hierarchy [13]. The AHP requires minimal mathematical calculations and is the only methodology that can consider consistency in decision-making [14]. In addition, it has been applied in construction industry to select suppliers [15], construction method [16], and equipment [17]. The general AHP procedure is described in Figure 2 [10].

The hierarchy levels are structured in such a way that there is a set of alternatives at the minimal level and a general goal is placed at the top level. In between the minimal and top levels, the general criteria and sub-criteria are placed [18]. After this, logical hierarchy levels are constructed, and the decision-maker can systematically assess the alternatives based on pair-wise comparison judgments. The pair-wise comparisons are conducted using Saaty’s [10] predefined scale (generally a nine-point scale [Table 1]).

If there are *n* criteria, the pair-wise comparison of criteria *i* and *j* yields an (*n* × *n*) dimension matrix *A*, where $a_{ij}$ denotes the comparative importance of criterion *i* with respect to criterion *j*.

(1) $$A=\left[\begin{array}{ccc} a_{11} & \cdots & a_{1n}\\ \vdots & \ddots & \vdots \\ a_{n1} & \cdots & a_{nn} \end{array}\right],\;a_{ij}=1,\;when\;i=h\;and\;a_{ji}=\frac{1}{a_{ij}}$$

After the pair-wise comparison, relative weights of criteria can be computed. The computation also includes the calculation of a normalized principal eigenvector from the given matrix *A*. The relative weights are derived by the eigenvector (*w*) corresponding to the largest eigenvalue (*λ_max_*) such as:

(2) $$Aw=\lambda_{max}\times w.$$

The evaluation requires a certain level of matrix consistency and can be checked by the consistency index (*CI*) as follows.

(3) $$CI=\frac{\lambda_{max}-n}{n-1}$$

If the matrix is perfectly consistent, then *CI* = 0. Consistency is an important factor in AHP, so it must be checked for each pair-wise comparison matrix at each stage.

Finally, the consistency ratio (*CR*) can be calculated such as:

(4) $$CR=\frac{CI}{RI}.$$

The RI is a random index that can be derived from the number of criteria n. Usually, a CR of 0.1 (10%) or less is considered acceptable (enough to be trustworthy).

## 3. Case Study: Material Selection for System Form

Formwork has a direct effect on the quality of the concrete surface, concrete framework cost, noise level, environmental issues, labor productivity, and even worker safety, because it is labor-intensive and physically demanding work [19]. From among the different types of forms, in this paper, a system form was selected for case verification because it is the most widely used form in mid- or high-rise building construction around the world. The term system form denotes a standard prefabricated form unit that normally consists of a panel and inner and outer frames. The system form can secure a high-quality concrete surface and high productivity with more recycle times than conventional concrete form (e.g., hand-set wood form), so the material selection of the system form can be more critical for the improved performance of the total construction project. The scope of this case is to select materials for the panel, and inner and outer frames.

### 3.1. Prospective Materials

In the formwork materials market in South Korea, steel, aluminum, wood, magnesium, and titanium alloys are commercialized, and 13 engineering plastics (polymers) are available. Thus, the prospective materials in this case study were limited to these 18 materials. Each material has different characteristics in terms of mechanical, functional, and physical properties, and each of the properties has three different criteria (Table 2). These criteria may not be limited to formwork material selection, and they can broadly be applied to any other construction material selection. Allowable formwork material properties according to the performance criteria is provided in Table 2.

### 3.2. Translation

The starting point of material selection for a system form is to identify the performance goal. There are many different performance criteria for formwork, but in this study, the authors set the goal with respect to maximizing user requirements while satisfying the technical requirements of formwork.

#### 3.2.1. User Requirements (Performance Goal)

There is insufficient research on how to improve overall formwork performance [19]. This paper tried to test a form after constructing it in such a way that it satisfied the requirements of the users (i.e., workers and engineers) who use it in practice on a construction site. To derive the user requirements, interviews were first conducted with two supervisors of two high-rise building construction projects, four heads of formwork companies, and six experts in system formwork on-site. A detailed interview process is shown in the author’s previous work [19,20]. The results of these interviews are summarized in Table 3. In Table 3, the importance index is a value obtained by dividing the importance value by the current performance value by examining the users’ requirements with importance and performance. Therefore, as the number increases, the more important or urgent the issue to be improved is for increasing formwork performance.

#### 3.2.2. Function and Components

Apart from the performance goal presented, a form must be designed to meet the required technical performance level as a product. A system form can be defined as a temporary structure that helps to hold the fluid concrete in place until it hardens and acquires a particular shape [21]. The system form is dismantled after the finishing formwork, but its technical performance strongly affects the subsequent tasks with respect to cost and duration. Therefore, the system form should be well designed not only to create the rigid, strong conditions required during concrete casting to avoid loss of concrete or collapse, but also to secure high performance (e.g., high constructability and a smooth concrete surface).

**Figure 3:**
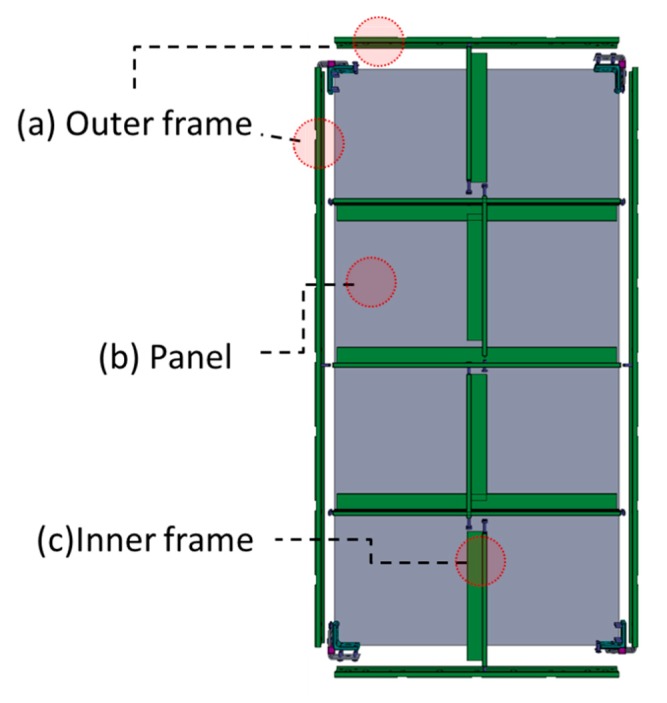
Basic concrete system form module used in South Korea. An outer frame (**a**), a panel (**b**), an inner frame (**c**).

The general system form is divided into an inner frame (Figure 3c), an outer frame (Figure 3a), and a panel (Figure 3b). The panel is in contact with the concrete and transfers the load (e.g., live load and wind load) to the inner frame. The inner frame receives the load from the panel, transfers it to the outer frame, and is installed at regular intervals to prevent deformation of the panel. The outer frame receives the load from the inner frame and delivers it to the shore or beam. Two or more different materials can be applied as composite (heterogeneous) materials in one concrete form because the technical requirements differ for each part in a system form (Table 4). For example, aluminum inner frames and steel outer frames with a wooden panel can be combined in a system form.

### 3.3. Screening

#### Constraints

A concrete form should endure concrete pressure, live load, and dead load; thus, during concrete casting, there are constraints in mechanical properties to avoid accidents caused by deflection. In addition, from previous research by the author [19,20], there are functional and physical requirements as well for a concrete form. If a material does not satisfy the requirements for each component (i.e., outer frame, inner frame, and panel), it is excluded from the list of alternative materials. Table 5 shows the constrains and possible materials for each form part. In this table, the moment of inertia (I) is assumed to be 6.0357 for the inner frame and 27.77 for the outer frame, considering the size of the normal Euro form, which is one of the most widely used system forms (600 mm × 1200 mm) in Europe and Asia. However, the required specifications differ between the manufacturers, countries, and times, so the material engineer should make constraints considering the potential users, purpose of the product, and performance goal.

### 3.4. Rating

In the rating procedure, the priority ranking is quantitatively calculated to improve user requirements according to the goal, criteria, and alternatives.

#### 3.4.1. Technical Performance Judgment for Each Alternative Material

There are four qualitative features in the evaluation criteria: impact resistance (IR), noise generation (NG), weather resistance (WR), and alkali resistance (AR). These features can only be evaluated relatively according to the performance goals. For example, in urban city projects, NG is very important because there are many residents near the construction site. In this situation, the designer may evaluate NG as a critical factor for performance. For this reason, these features should be evaluated by material designers or users according to their needs. In contrast, there are five quantitative features: flexural strength (FS), FM, density (DE), water absorption (WA), and TC. These features can be evaluated quantitatively according to the technical requirements of the project. In this study, 12 formwork experts simply evaluated them in the following manner: 7, 5, 3, and 1 point are given for excellent (S), good (A), fair (B), and poor (C) performance for qualitative features. In the case of quantitative features, the values of the properties were divided into four sections in the order of the highest values, and 7, 5, 3, and 1 are allocated, respectively. Based on this evaluation process, candidate materials in Table 6 were evaluated. The total summation of the scores from these four features is defined as the performance score (PS). The PS for nine quantitative and qualitative features are provided in Table 7.

The required characteristics may differ according to the form parts (i.e., inner frame, outer frame, and panel). For example, the noise generated during installation and dismantlement is crucial for the outer frame, but not for the inner frame, because it is not hit when dropping. AR is also more important for the outer frame and panel than for the inner frame because only a small amount of concrete sticks to the inner frame compared with the outer frame and panel. WR is crucial for the inner and outer frames because they are permanent-use parts exposed to outdoor conditions with wind and rain, but not for the panel because the latter is a disposable item that is replaced periodically.

#### 3.4.2. Material Selection Methodology Using AHP

**Figure 4:**
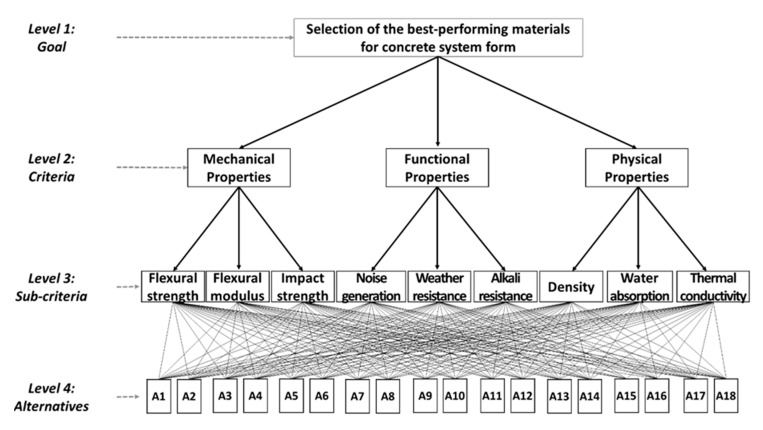
Hierarchical diagram for material selection criteria for concrete form.

Generally, AHP consists of three main principles, including the hierarchy framework, priority analysis, and consistency verification [10]. The first stage in applying the AHP method for material selection is to develop an AHP hierarchical framework that shows a systematic overview of goals, criteria, sub-criteria, and alternatives. The hierarchical framework looks like a tree from level-1, which represents the goal of selection, to level-2, which represents the criteria or factors that affect the goal, and level-3, which consists of the components of each criterion at level-2; their weights are quantitatively calculated to select optimal materials at level-4 (alternatives) (Figure 4). We referred the criteria and sub-criteria from Ashby’s [22] model that is one of the most widely used material design models.

At AHP hierarchy level-1, the goal of the project (the case study) is defined, and it is to select the most suitable materials hybridized with different materials for each component in the concrete form, outer frame, inner frame, and panel. Afterward, the structure is expanded to level-2, where the main criteria (mechanical, functional, and physical properties of the technical requirements) are represented. These are divided into several sub-criteria (FS, FM, IR, WR, AR, NG, DE, WA, and TC). Once a hierarchy framework has been constructed, users (i.e., workers and engineers) are requested to participate in a survey for a pair-wise comparison matrix at each hierarchy. In the priority analysis stage, each comparison matrix is calculated by an eigenvector to determine the weight of each criterion and the performance of alternatives [23]. The final stage is to calculate a CR to measure the consistency of the judgments in the survey. This is a comparison between the CI and random consistency index (RI). AHP allows assessment inconsistencies but they should not exceed 10%.

**Figure 5:**
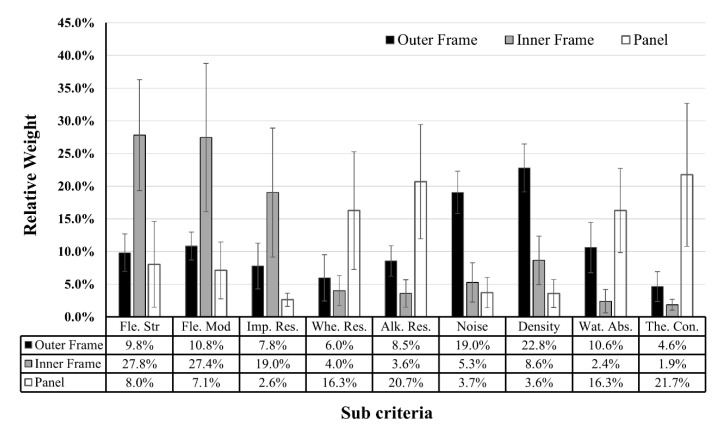
Overall relative weights of concrete form materials.

The AHP questionnaire was a pair-wise comparison of the questionnaires for each component from a total of 10 formwork experts (five engineers, five users, with 18 years of experience on average). For the outer frame, the λ value was 9.491 and the CR value was 4.2%. For the inner frame, the λ value was 10.084 and the CR value was 9.4%. For the panel, the λ value was 10.263 and the CR value was 9.1%. Figure 5 shows the overall relative weights for each part of the concrete form for nine sub-criteria. This relative weight, which is the priority value (w) of each material, can be calculated to select the optimum material for each constituent member of concrete form in terms of the performance goal.

#### 3.4.3. Best Materials Choice

The priority index (PI), which is defined as the priority value (w) × PS divided by cost, is newly suggested for quantitative comparison among materials. The PI quantifies the effect of material properties on a user’s performance goal. In other words, a material that has a high PI should be selected to optimize the performance of materials. The PI can be designed and defined differently depending on the design goals. Table 7 shows the calculated PI for the outer frame, inner frame, and panel. In addition, the disqualified and highest score alternatives are selected.

## 4. Development of Composite System Form

**Figure 6:**
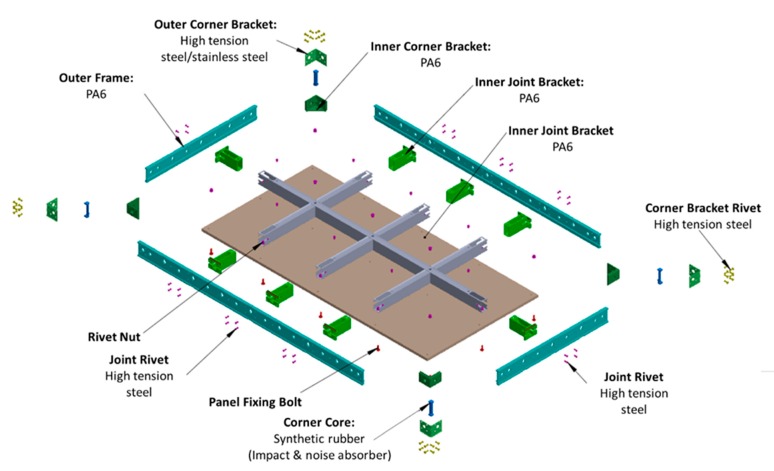
Detailed configuration of the composite system form (CSF).

After the material selection process, the detailed configuration of a new composite system form (CSF) was drawn (Figure 6). The highest PI materials for the outer frame, inner frame, and panel were polyamide 6 (PA6), steel, and polypropylene (PP), respectively. The connecting bracket between the members was made of PA6, and the rivets and bolts were made of steel. In the case of the panel, a PP sandwich panel was used, but for the low FM value of PP and the ease of nailing, a second prior material, plywood coated with PP or high-pressure laminate (HPL) film was also tested. The design of the inner frame, outer frame, and panel is described in more detail in the following section.

### 4.1. Material Selection for Each Part of the CSF

#### 4.1.1. Outer Frame

The outer frame is in direct contact with the concrete and is exposed to the weather and external impacts. Therefore, it should be alkali and weather resistant with low adhesion and high IR to withstand impact during installation and dismantlement. There are two alternative outer frame materials, namely, plastics and alloys. Given that there are various kinds of plastics, among the materials satisfying the mechanical requirements in Table 6 and Table 7, PA6 has the highest PI value. In this study, PA6 was selected as the outer frame material considering the PI value, but it may be changed at any point based on the material cost. PA6 does not shrink, is resistant to ultraviolet (UV) radiation, and is insensitive to knocks and scrapes. In addition, even if some concrete sticks to the frame, it is easy to clean because it does not react with concrete.

#### 4.1.2. Inner Frame

As shown in Table 5, the possible materials for the inner frame are aluminum, steel, alloys, and plastic. Compared with an external frame, there are many alternatives for the inner frame because it has less contact with concrete or weather, and the possibility of external impact is low. Considering the PI, the best material is steel. Because the strong modulus of elasticity of steel prevents deflection, it is the most suitable material for the inner frame, which is most affected by deflection, at a comparatively low price.

#### 4.1.3. Panel

The panel material should be plywood or plastic, as shown in Table 5, as it should be lightweight and capable of being released from concrete even when no release agent is applied. Considering the PI, PP ranked the highest; however, because of the weak FM of PP and the difficulty of nailing, we tested both sandwich panels of PP and HPL-coated wood panel, which are on the second tier on PI for panels. These special panels ensure chemical resistance, moisture resistance, and UV resistance.

#### 4.1.4. Corners and Brackets

PA6 was used for the joint brackets, and synthetic rubber was applied for the core corners because it can reduce noise as well as impact damage during installation and dismantling, which were strong constraints for all assembly parts. In addition, the CSF, which is assembled from several separable structures, has advantages as a temporary resource because it is easily replaced part-by-part when damaged.

### 4.2. Verification of the CSF

After designing the product with the selected materials and applying the design to the actual site, we compared and analyzed the performance of the CSF versus existing forms. There are many performance criteria, but the authors validated only the recycle time, NG, and work efficiency because these are the three most important performance criteria for concrete formwork.

#### 4.2.1. Three-Dimensional (3D) Modeling of the CSF

**Figure 7:**
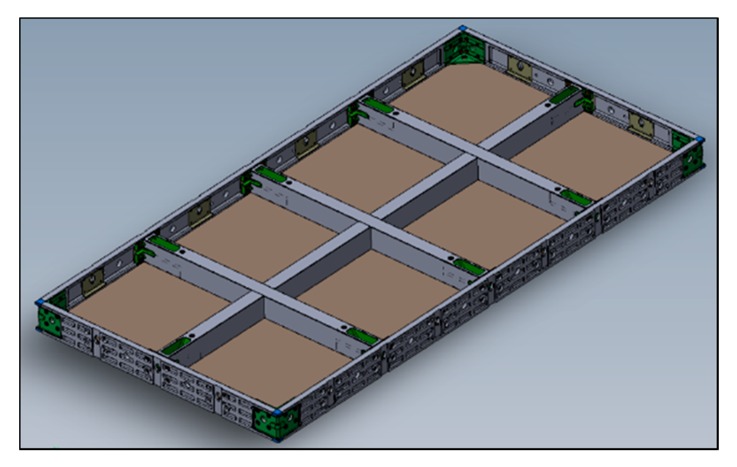
Three-dimensional model of the CSF.

After checking the structural analysis, a detailed 3D model of the CSF was drawn for the purpose of fabricating real prototypes (Figure 7). Through computer analysis, the frame structure was optimized to minimize the amount of material input and deflection.

#### 4.2.2. CSF Prototype

**Figure 8:**
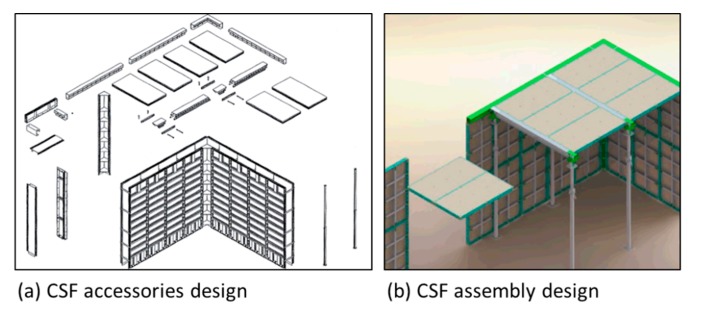
The CSF prototype design ((**a**) accessories components design, (**b**) assembly method design).

**Figure 9:**
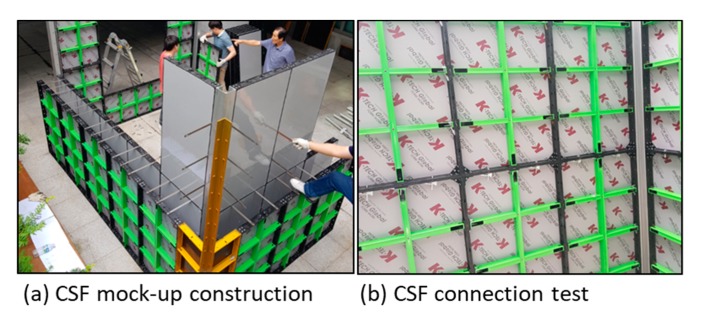
A mock-up assembly house made up of CSF ((**a**) constructability test, (**b**) connectivity test).

After the 3D design of the CSF, a prototype was developed including the panel, corner panel, and accessories to build a mock-up model house (Figure 8). Installation and assembly tests were then conducted to check the applicability of the prototype (Figure 9).

#### 4.2.3. Structural Analysis (Standard Loading Procedure)

**Figure 10:**
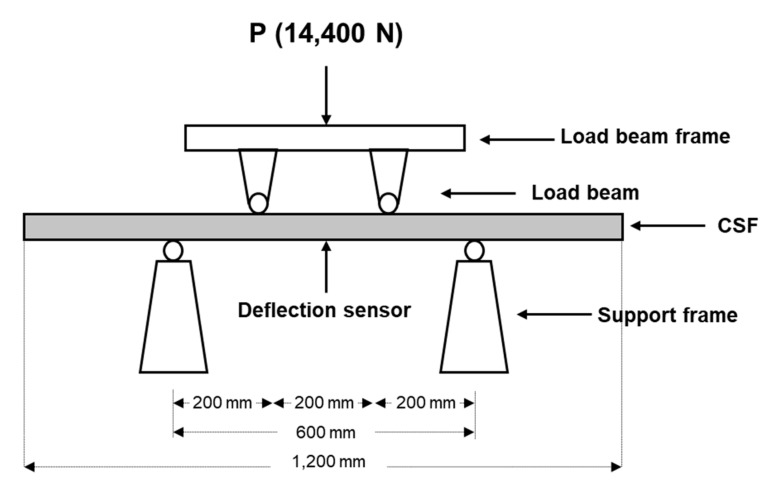
KS F 8006 (standard loading method for concrete form in Korea).

A standard loading test was performed on the fabricated CSF prototype to calculate the deflection of the form during concrete casting. Because there is no official performance standard for composite concrete forms in Korea, the loading was performed based on the Korea Standard (KS) criterion F 8006 (Figure 10) with a maximum load (P) of 14,400 N. KS F 8006 is a very strong standard for steel form, and if the maximum deflection is less than 1.4 mm, it ensures that the form is strong enough to endure concrete pouring in any position. The CSF deflection did not exceed 1.4 mm during 14,400 N loading, verifying the safety of the CSF during concrete casting.

#### 4.2.4. Recycle Time Test

The CSF consists of an assembly structure (i.e., panel + frames), and the panel can be changed if it is worn out or damaged. Current plywood, the most widely used formwork panel, can be reused (recycled) 7 to 10 times without replacement. The panel replacement is a cost- and time-consuming task, so a higher recycle time ensures better formwork performance. In this study, recycle time tests were performed for the PP sandwich panel and HPL-coated wood panels. Because there is no official certification test for the number of times a form can be reused, the evaluation was performed subjectively by comparing the change to the surface of the panel with the surface of the concrete after removing the concrete. After running a concrete casting test 50 times without changing the panel, the surfaces of the CSF and the concrete remained clean, even without cleaning, because the concrete did not stick to the PP or thin film-covered panel. In addition, the new panels are neither worn out or damaged by concrete because they have enough IR and AR. This result means that the CSF is suitable for high-rise building construction requiring the repeated use of panels.

#### 4.2.5. Noise Test

**Figure 11:**
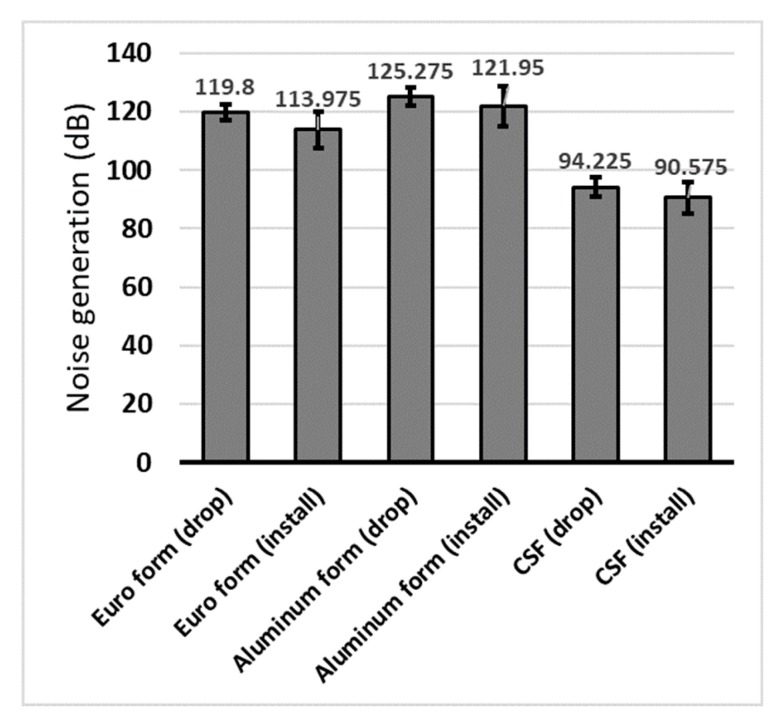
Test result of noise generation (NG) during dropping and installation.

Two kinds of tests were conducted to measure the noise produced when using the CSF, generated when dropping the CSF from a certain height to the floor, and generated while installing and dislodging pins with a hammer. The average value was measured after four tests using a noise meter. For comparison with existing forms, the same experiment was performed on the Euro and aluminum forms, and the measurement results are shown in Figure 11. The CSF dampens noise through shock absorption and the separate frame structures, resulting in less noise than other forms.

Table 8 shows a comparison of the characteristics of the proposed CSF with existing aluminum and Euro forms.

#### 4.2.6. Field Application

**Figure 12:**
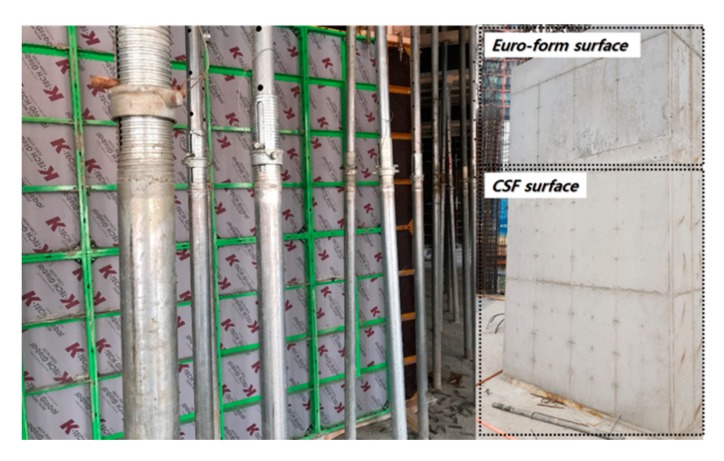
Concrete surface quality comparison between the CSF and the Euro form.

The developed CSF was applied to an actual building construction site to validate the performance and quality of the CSF. The average formwork time between the aluminum form and CSF were compared and analyzed during a task of wall, slab, and stair formwork (Table 9). Each of the formwork tasks consists of four work tasks: stripping, lifting, plaster form oil, and installation. The average task (i.e., installation and dismantlement) time of 20 units of CSF and Al-form were measured. In addition, to validate the concrete quality, CSF was applied alongside the existing Euro form (plywood panel), and the concrete surface quality was compared and analyzed after stripping forms (Figure 12).

## 5. Results

Figure 5 shows the overall relative weights of the material performances. The authors calculated the PI by multiplying the material property data in Table 6 by the relative weights of the performances. Table 7 shows the results of PI for each material and allowable candidates, considering the technical requirements. The outer frame has higher importance for NG and DE, the inner frame has higher importance for FS and FM, and the panel has a higher importance for TC and AR. When considering the PI, PA6, steel, and PP have the highest values for the outer frame, inner frame, and panel, respectively, but PP has several limitations on deflection and is difficult to nail. The HPL-coated plywood panel is the second-highest alternative for the panel, so we tested it with PP panel during the field test.

A new composite system form (CSF) was developed based on the result of the case study, and the authors verified its structural safety and then tested applicability in terms of the panel’s recycle time, NG, and work efficiency, and these are the most important factors for formwork performance.

In the developed CSF, PP- and HPL-thin film coated on the plywood panel adds impermeability and does not stick to concrete, and they can be reused more than 50 times without applying form oil. In addition, the noise level of the CSF was lower than 95 dB during the installation and dismantling work, a remarkable reduction compared with the aluminum (121.95–125.275 dB) and Euro (113.975–119.8 dB) forms. Most importantly, CSF was 33% lighter than the conventional aluminum form (15 kg) and 47% lighter than the Euro form (19 kg). The weight reduction, compared with the aluminum form, provided a 27.5% increase in work productivity during wall, stair, and slab formwork. In addition, CSF shows 100% compatibility with Euro form and Al-form, and it can be systemized as Table form or Gang form by assembly. 

## 6. Discussion

This paper aims to develop an MCDM technique for the construction material selection model to help researchers and practitioners select optimal materials in terms of their performance goals. In this regard, this study proposed an AHP-based MCDM procedure for construction materials and provided a case as a guideline for further applications. A newly developed CSF is structurally safe during and after concrete casting because it passed the KS standard. This does not simply describe safety as a small-scale form; the same structure of the form module can be assembled into a large size and various shapes of concrete forms in building construction such as gang form and slip and truss tables.

The result of the recycle times shows that the CSF panel and the PP- and HPL-thin film coated on the plywood can be reused more than 50 times without applying form oil. Replacing the formwork panel is very costly and time-consuming work especially in mid- and high-rise building construction. In particular, when constructing irregularly shaped buildings, the installation location of the form is fixed, and it is very inefficient work to replace the formwork panel. In this respect, the suggested CSF panel can be an innovative and state-of-the-art technology to reduce formwork productivity (e.g., duration and cost).

In contrast, CSF reduced NG during the formwork process by up to 30 dB compared with aluminum form. Consequently, the amount of noise generated is reduced by 1000 times. The NG of the formwork is the main cause of complaints around the construction site, along with the hearing damage of workers. This often lowers the productivity of the construction projects and creates a bad social perception, in turn inhibiting the influx of skilled workers. Therefore, reducing noise can play a very important role in increasing the productivity and sustainability of construction in the construction industry.

The CSF was 33% lighter than the conventional aluminum form (15 kg) and 47% lighter than the Euro form (19 kg). It helped reduce formwork time by 27.5% by increasing the formwork performance. Because the formwork is transported, installed, and dismantled by a worker, its weight has a significant impact on performance. Weight reduction can also play a major role in reducing the risk of safety accidents caused by formwork dropping and the operator’s physical fatigue. In addition, because more forms can be loaded on a truck at once, transportation costs can be reduced. Thus, using the CSF not only saves on cost, but also reduces construction time.

Finally, CSF is 100% compatible with aluminum form and Euro form because it is designed with high compatibility, so that workers may not be confused when they work with this new system form. This is significant in construction sites where very large amounts of forms are required. The formwork company can use the part where new material needs to be applied optionally without having to purchase all CSFs covering the total formwork area.

These verifications show that the optimal choice of materials in the field of building construction could have an effect on the construction methods and could even change the technical paradigm of the construction as a whole.

## 7. Conclusions

This study proposed a material selection model for construction materials and applied the model to a concrete form, a key temporary resource in building construction. The CSF consists of several separately designed parts and each component has different technological requirements with respect to properties such as IR, WR, and AR. As each index is related to a specific performance parameter (e.g., productivity, concrete surface quality, and corrosion), careful selections should be made according to user requirements. Using a systematic and scientific design through AHP methodology, a highly advanced concrete form was fabricated that satisfied both the user requirements and the technical requirements of the system formwork for performance improvement.

The results of this study suggest that the appropriate selection of construction materials is very effective as a method for increasing construction performance. Moreover, problems that involve productivity decrease, and safety accidents and environmental damage can be addressed; such issues have been identified in the construction field as being in need of improvement with the supplementing of materials that have been made in an empirical and intuitive manner.

Several limitations of this study should be addressed in future work. First, the cost parameter considered was only that of raw materials without the processing and recycling costs. Second, there were not enough people to be surveyed on AHP, so more practitioners should have participated for accurate and general implications. In addition, it was difficult to apply the new concrete form because several construction companies declined to apply the CSF, which was not verified earlier. Third, the shape optimization of the CSF frame was insufficient. In future research, a topological optimization method for designing an optimal CSF shape that satisfies the demand load condition should be explored. In a further study, more generalized functions and validation should be provided.

## Figures and Tables

**Table 1:** Nine-point scale suggested by Saaty [10].

Definition	Intensity of Importance
Equally important	1
Moderately more important	3
Strongly more important	5
Very strongly more important	7
Extremely important	9
Intermediate values	2, 4, 6, 8

**Table 2:** Criteria for material selection of concrete form.

Criterion	Properties	Explanation
Flexural strength (FS)	Mechanical	Because the formwork must withstand the loads of concrete, the minimum FS required by the design must be secured. Generally, when the concrete, live, and dead loads are applied, the minimum FS value is set so that it is below the allowable deflection.
Flexural modulus (FM)		The higher the FM, the better the quality of the concrete surface, as the deflection of the form decreases as the concrete is poured.
Impact resistance (IR)		Strength to withstand breakage of the form when dismantling and dropping of formwork. It should secure sufficient strength to prevent breakage.
Weather resistance (WR)	Functional	It should not be deformed or corroded by weather such as ultraviolet rays, snow, or rain.
Alkali resistance (AR)		Because the concrete exhibits strong alkaline properties, the material in contact with the concrete must have AR.
Noise generation (NG)		Noise created during the installation, disassembly, and dropping of the formwork causes psychological damage to the operator and the site, so it is necessary to use a material with low noise.
Density (DE)	Physical	During installation and disassembly, forms are carried by the workers, so the lowest density material should be used to reduce the weight as much as possible. Reducing the weight of the form not only increases the productivity and constructability of formwork, but also reduces the incidence of work accidents.
Water absorption (WA)		Forms should be made of a material that absorbs as little moisture as possible because they are continuously exposed to a wet environment and affected by rain.
Thermal conductivity (TC)		To achieve uniform quality in the curing process during hot and cold weather, materials that come into contact with concrete should have low TC. In addition, materials with low TC are particularly important when not using a release agent because they have an advantage for making relatively smooth surfaces.

**Table 3:** User requirements for form.

No.	Category	User Requirements	Importance Index	Rank
1	Constructability	Easy assembly and disassembly (can be fit and fastened together with reasonable ease)	91.3	1
2		Low noise during dismantlement or assembly and disassembly	87.4	2
3		Easy separation from concrete	76.2	8
4		Efficient lifting and carrying	79.6	6
5	Safety	Not distorted or deflected during concrete casting	56.3	15
6		Reduced work accidents (struck by object)	57.7	13
7	Durability	High repeat use with constant module size	83.2	4
8		Recyclable material usage	61.4	12
9		Durable against falling and external impacts	64.2	10
10		Easy maintenance and cleaning	69.9	9
11	Reliability	Low TC (low temperature sensitivity)	59.1	14
12		High concrete surface quality	78.8	7
13	Conformance	Compatible (size, height, fixing method) with existing formwork units (e.g., Euro form, aluminum form, and Skydeck)	86.7	3
14		Hybrid (concurrent usage) usage for vertical (wall and column) and horizontal (slab) forms	63.1	11
15		Provides various module sizes to minimize on-site work (filler and conventional formwork)	81.9	5

**Table 4:** Technical requirements for form materials.

Factors for Consideration	Inner Frame	Outer Frame	Panel
Mechanical consideration	High FS ^a^ and FM ^b^	High FS and FM, high IR ^c^	High FM, high IR
Functional consideration	Reduced noise, WR ^d^	Reduced noise, continuous use temperature, AR ^e^, WR	Reduced noise, low TC ^f^, easy to nail, easy to change, easy to strip off concrete, AR
Physical consideration	Lightweight, low WA ^g^	Lightweight, low WA	Lightweight, low WA

^a^ Flexural Strength, ^b^ Flexural Modulus, ^c^ Impact Resistance, ^d^ Weather Resistance, ^e^ Alkali Resistance, ^f^ Thermal Conductivity, ^g^ Water Absorbtion.

**Table 5:** Possible materials for each form part.

Properties	Inner Frame	Outer Frame	Panel
Functional requirements (strong constraints)	NG ^a^ ≥ B WR ^b^ ≥ B	NG ≥ B WR ≥ A AR ^c^ ≥ A	NG ≥ A Easy to nail
Mechanical requirements (strong constraints)	FS ^d^ > 75 Mpa for the wall, 45 Mpa for the slab FM ^e^ > 167,751 kgf/cm^2^ for wall, 95,059 kgf/cm^2^ for slab If I is 6.0357	FS > 64 Mpa for the wall, 42 Mpa for the slab IR ^f^ ≥ A FM > 218,761 kgf/cm^2^ for wall, 123,965 kgf/cm^2^ for slab If I is 27.77	FS > 64 Mpa for the wall, 42 Mpa for the slab IR ≥ A FM > 73,242 kgf/cm^2^ for wall, 41,504 kgf/cm^2^ for slab
Physical requirements (weak constraints)	WA ^g^	DE, WA	DE, WA, low TC ^h^
Possible materials (weak constraints)	Aluminum, steel, plastic, alloys	Plastic, alloys	Plywood, plastic

^a^ Noise Generation, ^b^ Water Resistance, ^c^ Alkali Resistance, ^d^ Flexural Strength, ^e^ Flexural Modulus, ^f^ Impact Resistance, ^g^ Water Absorption, ^h^ Thermal Conductivity.

**Table 6:** Allowable formwork material properties according to the performance criteria.

Materials	Flexural Strength (FS) (MPa)	Flexural Modulus (FM) (GPa)	Impact Resistance (IR)	Weather Resistance (WR)	Alkali Resistance (AR)	Noise Generation (NG)	Density (DE) (kg/m^2^)	Water Absorption (WA) (%)	Thermal Conductivity (TC) (W/(m⋅K))	Price (€/kg)
Steel	400	210	S	B	A	B	7850	-	45	0.45
Aluminum	386	70	S	A	C	C	2712	-	205	1.95
Wood (oak)	60	11	C	C	C	S	650	> 8	0.16	0.35~0.9
Magnesium alloys	150	45	S	B	A	B	1738	-	165	5~15
Titanium alloys	800	110	S	B	A	B	4500	-	15	15~20
CFRP^1)^	900	89	S	A	A	A	1550	-	0.5–3.0	13~22
ABS^2)^	75–128	2.5–8	S	C	A	A	1070	0.3	0.1	2.05
Acetal (POM)^3)^	85	2.5–11	A	C	A	A	1410	0.25	0.22	0.7
PVC^4)^	35	3.1–8	C	A	A	A	1470	0.06	0.19	0.95
Nylon 6 (PA6)^5)^	85–405	2.4–20	A	A	A	A	1130	1.2	0.25	1.8
PA66^5)^	103–420	3.1–18	A	B	A	A	1183	1.2	0.26	1.92
Polyimide	175	5–32	A	S	A	A	1420	0.2	0.11	3.5
Polycarbonate	90–138	2.3–4.4	S	A	C	A	1200	0.15	0.20	2.8
Polyethylene	40	0.7–6	S	B	A	A	970	0.01	0.11	0.8
PET^6)^	80	1	A	A	A	A	1380	0.1	0.15	1.4
PBT^7)^	79–270	2.6–13	A	A	A	A	1310	0.08	0.29	2.95
Polypropylene	40–190	1.5–8	A	A	S	A	946	Slight	0.12	0.7
Polystyrene	70	2.5–13	A	B	A	A	1040	-	0.11	0.76

S: Excellent; A: Good; B: Fair; C: Poor. CFRP^1)^: carbon fiber reinforced plastic; ABS^2)^: acrylonitrile butadiene styrene; POM^3)^: polyoxymethylene; PVC^4)^: polyvinyl chloride; PA6, PA66^5)^: polyamides 6, 66; PET^6)^: polyethylene terephthalate; PBT^7)^: polybutylene terephthalate.

**Table 7:** Scoring for priority index (PI) using material properties and relative weights of performances.

Materials	FS	FM		IR	WR		AR	NG		DE	WA	TC	PI for the Outer Frame	PI for the Inner Frame	PI for Panel
Steel	7	7		7	3		5	3		1	7	1	970.9	840.4	1072.4
Aluminum	7	7		7	5		1	1		1	7	1	209.1	154.6	241.2
Wood (oak)	1	5		1	1		1	7		7	1	5	563.9	553.9	552.3
Magnesium alloys	5	7		7	3		5	3		1	7	1	42.5	37.0	45.0
Titanium alloys	7	7		7	3		5	3		1	7	1	25.0	21.6	27.6
CFRP^1)^	7	7		7	5		5	5		3	7	3	30.5	29.1	32.2
ABS^2)^	3	3		7	1		5	5		7	3	7	243.4	196.1	206.3
Acetal (POM)^3)^	3	3		5	1		5	5		3	3	5	515.0	504.6	511.1
PVC^4)^	1	3		1	5		5	5		3	5	5	354.6	461.5	390.9
Nylon 6 (PA6)^5)^	7	5		5	5		5	5		7	1	3	280.8	284.3	239.6
PA66^5)^	7	5		5	3		5	5		5	3	3	239.3	231.6	230.4
Polyimide	5	7		5	7		5	5		3	3	7	137.3	155.4	159.8
Polycarbonate	3	1		7	5		1	5		5	1	5	149.3	143.8	113.0
PE^6)^	1	1		7	3		5	5		7	5	7	640.9	558.0	494.8
PET^7)^	3	1		5	5		5	5		5	5	7	330.5	343.9	305.7
PBT^8)^	5	5		5	5		5	5		5	5	3	166.2	168.2	154.8
PP^9)^	5	1		5	5		7	5		7	7	7	795.9	784.9	722.3
Polystyrene	1	3		5	3		5	5		7	1	7	591.2	570.3	479.7

CFRP^1)^: carbon fiber reinforced plastic; ABS^2)^: acrylonitrile butadiene styrene; POM^3)^: polyoxymethylene; PVC^4)^: polyvinyl chloride; PA6, PA66^5)^: polyamides 6, 66; PE^6)^: polyethylene; PET^7)^: polyethylene terephthalate; PBT^8)^: polybutylene terephthalate; PP^9)^: polypropylene; 
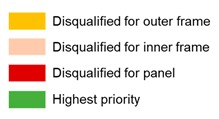
.

**Table 8:** Comparison of characteristics of the CSF and existing forms.

Item	CSF	Aluminum Form	Euro Form
Material	Composite (PA6 GF60) + Steel + HPL-coated plywood panel	100% Aluminum	Steel + coated plywood
Image	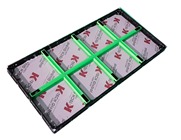	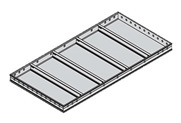	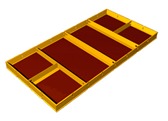
Specification	600 mm × 1200 mm	600 mm × 1200 mm	600 mm × 1200 mm
Weight	10 kg	15 kg	19 kg
Number of recycling cycles	Above 50 times	Above 50 times	Below 10 times
Use of form oil	None	Use	Use
Noise creation	Below 95 dB	Above 120 dB	Above 110 dB
Applicability	Wall + Slab	Wall + Slab	Wall
Systemization	Table form, gang form	-	-
Compatibility	100% compatible with both Aluminum and Euro forms	Not compatible with Euro form	Not compatible with Aluminum form

**Table 9:** Performance comparison between CSF and Al-form.

Task Distribution		Avg. Task Time (Al-form) (s)	Avg. Task Time (CSF) (s)
Wall formwork	Stripping	30	26
	Lifting	21	16
	Plaster form oil	10	-
	Installation	48	34
Stair formwork	Stripping	32	27
	Lifting	16	12
	Plaster form oil	10	-
	Installation	54	39
Slab formwork	Stripping	18	17
	Lifting	14	14
	Plaster form oil	10	-
	installation	24	22
Total		287	208
Measurement time range			
**Stripping**: (start) When the tools for dismantling begin to reach a form; (end) when the dismantled form is placed on the slab.			
**Lifting**: (start) When workers started to hold the form to lift; (end) when putting down the lifted form in the work area.			
**Plaster form oil**: (start) When workers start to grip the roller and take form oil; (end) when the roller is put down after plastering.			
**Installation**: (start) When workers start to hold the form by hand; (end) when the tool is put in after installation.			

## Data Availability

All data, models, and code generated or used during the study appear in the submitted article.
